# The Correlation between Malocclusion and Body Posture and Cervical Vertebral, Podal System, and Gait Parameters in Children: A Systematic Review

**DOI:** 10.3390/jcm13123463

**Published:** 2024-06-13

**Authors:** Dorota Różańska-Perlińska, Małgorzata Potocka-Mitan, Łukasz Rydzik, Patrycja Lipińska, Jacek Perliński, Norollah Javdaneh, Jarosław Jaszczur-Nowicki

**Affiliations:** 1 Medical Department, The Academy of Applied Medical and Social Sciences, 82-300 Elblag, Poland; dorotka@gumed.edu.pl (D.R.-P.); jacekperlinski@wp.pl (J.P.); 2 Institute of Humanities, Social Sciences and Tourism, Podhale State College of Applied Sciences in Nowy Targ, 34-400 Nowy Targ, Poland; potockamitan@interia.pl; 3 Institute of Sports Sciences, University of Physical Education, 31-571 Krakow, Poland; 4 Institute of Physical Education, Kazimierz Wielki University in Bydgoszcz, 85-064 Bydgoszcz, Poland; patrycja.lipinska@ukw.edu.pl; 5 Department of Biomechanics and Sports Injuries, Kharazmi University of Tehran, Tehran 14911-15719, Iran; njavdaneh68@gmail.com; 6 Department Physiotherapy, School of Public Health, Collegium Medicum, University of Warmia and Mazury, 10-719 Olsztyn, Poland

**Keywords:** malocclusion, body posture, head posture, podal system, gait parameters

## Abstract

**Background**: This study investigates the relationship between malocclusion and body posture, head posture, podal system, and gait parameters in children. **Methods**: A systematic review of observational studies from 2010 to 2023 was conducted and 24 cross-sectional studies involving 6199 participants were identified. These studies were categorized into those dealing with body posture (10 studies, 3601 participants), cervical vertebral column and head posture (6 studies, 644 participants), the podal system (5 studies, 1118 participants), and gait (3 studies, 836 participants). **Results**: Evidence suggests a significant association between malocclusion and body posture, balance, podal system, and gait parameters. Notably, eight studies found a significant relationship between malocclusion and body posture, while five studies identified this relationship with the cervical vertebral column and head posture, five with the podal system, and three with gait parameters. **Conclusions**: Overall, the quality of evidence was strong for the association between malocclusion and body posture and the podal system and moderate for head posture and gait parameters. These findings offer insights for therapists to design interventions tailored to children with malocclusion based on considerations of body posture, head posture, podal system, and gait parameters, though further longitudinal cohort studies are needed for better predictive understanding.

## 1. Introduction

The fundamental ability to control the body’s position in space is crucial for any action taken by humans, as all tasks need to meet specific postural demands. Various elements can influence body posture and balance, with dental malocclusion holding significant importance. It is widely recognized that stomatognathic system problems can influence overall well-being by causing chronic medical diseases [1], cardiovascular diseases [2], kidney malfunction [3], or even an increased risk of cancer mortality [4]. Contrariwise, good general health can positively affect dental health [5]. Manifestations of many systemic diseases can be found in the oral cavity, e.g., Sjogren’s syndrome [6], diabetes [7], or osteoporosis [8]. Although it has been established that dental health has an impact on general health if dentists are trained, not much emphasis is given to teaching them about the relationship between dental development, oral function, and posture [9]. On the other hand, the first descriptions of the correspondence between body posture and dental occlusion in children were published many years ago by Siedlecka-Głazik in 1978 [10] and Paphalmy in 1974 [11]. This initial approach formed a perspective on dental occlusion integrating with the broader structures of the human body and, since then, this subject has continued to captivate the attention of numerous scientists and clinicians.

The relationship between body posture and occlusal conditions can be explained with a mechanistic interpretation of postural compensations in the human body. “If one considers the human body as a closed mechanical system, the genetic make-up of an individual will dictate their body shape and musculo-skeletal composition. If there are any imbalances, for example, if there is a problem with one of the joints, there will be some sort of compensation to maintain the overall stability of the system” [9]. Pursuing this direction, alterations in head position observed in dental malocclusions are the catalyst for shifts in body balance, affecting the neck, vertebral column, and pelvis, as well as the positioning of the legs and feet. This conceptual framework may engender an innovative paradigm for clinicians and extend the scope of malocclusion treatment to encompass the correction of postural defects and the incorporation of physiotherapy interventions. Existing literature has documented the efficacy of physiotherapy in addressing malocclusion, as evidenced by reports in the field [12,13].

The sustained scholarly interest and significance attributed to the correlation between malocclusion and posture have spurred extensive research endeavors. Multiple literature reviews have been conducted to systemically organize the wealth of knowledge amassed in this domain [14,15,16,17,18,19]. Nevertheless, there exists a dearth of literature reviews specifically focused on the interplay between body posture and malocclusion in children. Considering that the most dynamic physiological changes occur during the growth phase, the decision was made to conduct a comprehensive review specifically addressing the relationship between body posture and occlusion in adolescents. The authors of the research posit a hypothesis suggesting that altering body posture through physiotherapy and physical exercises might prove notably efficacious in treating occlusal conditions during the intensive growth phase in children. The results of this study can be used in planning, management, rehabilitation, and the treatment of malocclusion.

## 2. Materials and Methods

The review followed the guidelines set by the Preferred Reporting Items for Systematic Reviews and Meta-Analyses (PRISMA) statement [20]. This systematic review was retrospectively registered in the PROSPERO database with ID CRD42024545531.

The investigation entails a systematic review executed by exploring primary articles within databases, including PubMed, Semantic Scholar, Medline, Scopus, Science Direct, and EBSCO. Search strategies varied for each database to account for different search capabilities. Additional articles were obtained from a list of references in published reviews. The reference lists of retrieved articles and grey literature were searched to detect studies potentially eligible for inclusion. All studies were evaluated for eligibility based on the following PICO model: (P) patients diagnosed with malocclusion without a history of orthodontic treatment were included; (I) not applicable; (C) the group of Class I malocclusion (subjects with a normal sagittal jaw relationship) was applied as the control to assess the relationship of body posture and cervical vertebral, podal system, and gait parameters with Class II and III malocclusion; (O) Outcome measures consisted of craniovertical angles, cervicohorizontal angles, craniocervical angles, cervical curvature, kyphosis angle, scoliosis angle, lordosis angle, and gait and podal system parameters. Search queries incorporated keywords such as “body posture and malocclusion”, “body balance and malocclusion”, “gait and malocclusion”, and “podal system and malocclusion”. Studies were identified by using all possible combinations of the following groups of search terms: body posture AND malocclusion, body balance AND malocclusion, gait AND malocclusion, podal system AND malocclusion, body posture AND occlusion, body balance AND occlusion, gait AND occlusion, podal system AND occlusion, scoliosis AND malocclusion OR occlusion, kyphosis AND malocclusion OR occlusion, lordosis AND malocclusion OR occlusion, head forward AND malocclusion OR occlusion, asymmetrical spinal AND malocclusion OR occlusion, cervical vertebral column AND malocclusion OR occlusion, head posture AND malocclusion OR occlusion, neck AND malocclusion OR occlusion, children AND malocclusion OR occlusion, static balance AND malocclusion OR occlusion, dynamic balance AND malocclusion OR occlusion, foot posture AND malocclusion OR occlusion, plantar pressure distribution AND malocclusion OR occlusion, center of gravity AND malocclusion OR occlusion, and gait parameters AND malocclusion OR occlusion. The inclusion criteria encompassed studies conducted in English from 2010 to 2023. Exclusion criteria involved research investigations deviating from the designated thematic scope. Furthermore, systematic review articles and those concentrated on adult populations were systematically excluded from the analytical framework. The research, adhering to predefined criteria, underwent a meticulous analysis encompassing the objectives, methodologies, outcomes, and conclusions within the designated field of study. A strategic decision was then reached to divide the forthcoming review into four discrete segments, each elucidating distinct keywords or core themes significant to the scrutinized investigations, namely: posture and balance in general, head posture and cervical vertebral column, podal system, and gait.

### 2.1. Study Selection

Initial screening of titles and abstracts was performed by two authors. The full texts of the included manuscripts were then reviewed by the same authors. In cases of disagreement, a third author was consulted to reach a consensus. At this stage, study designs, studies including chronic neck pain, those on how to measure fear of movement, the language of the articles, and other inclusion and exclusion criteria were reviewed (Table 1). The consensus of two out of three referees was considered as the decisive voice.

### 2.2. Data Extraction

The following relevant data were extracted by two authors from each study: first author with the year of publication, sample size, mean age, study design, statistical analysis methods, and outcome measurement. If necessary, the corresponding author of the study was contacted to obtain more information.

### 2.3. Risk of Bias Assessment

Two reviewers independently evaluated the risk of bias in the studies included in the review, using the (Newcastle–Ottawa Scale (NOS), http://www.ohri.ca/programs/clinical_epidemiology/oxford.asp, accessed on 25 April 2024) as a recommended tool for assessing the risk of bias in nonrandomized studies [21,22]. it is validated for case-control and longitudinal studies [23]. This tool assesses four key areas of risk of bias: selection bias (how study participants are chosen), performance bias (how confounding factors are managed), detection bias (statistical methods used), and information bias (how exposure and outcomes are measured). This study evaluated four domains using seven items, each of which was scored on a scale from 0 (high risk) to 3 (low risk). A total score of 0 to 6 indicated a high risk of bias, while scores of 7 to 13 and 14 to 21 indicated moderate and low risk of bias, respectively. The quality of the studies was assessed using the Modified Cochrane Back and Neck Group scoring system. Disputes were resolved through consultation or discussion with a third author.

A meta-analysis could not be carried out due to the presence of heterogeneity in terms of participant age, sample size, malocclusion condition, outcome measures, methods of measuring variables, and statistical methods used.

## 3. Results

**Figure 1:**
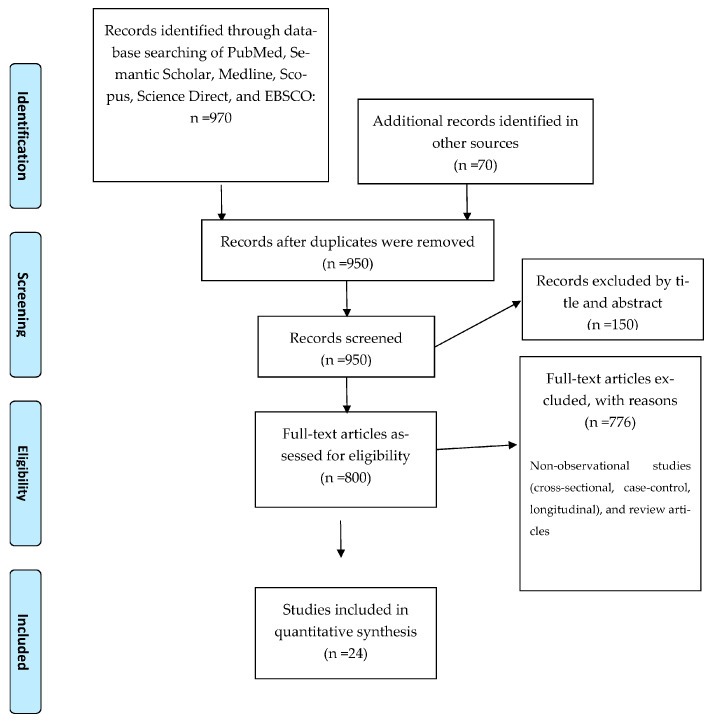
PRISMA chart for included studies.

After searching the databases, a total of 1050 articles were found. Of these, 24 cross-sectional studies with a total of 6199 participants met our inclusion criteria and were included in this review (Figure 1). In conclusion, we included a total of 24 research studies for analysis, distributed as follows: ten focused on body posture (3601 participants), six on the cervical vertebral column and head posture (644 participants), five on the podal system (1118 participants), and three on gait (836 participants). The results are summarized in Table 2, Table 3, Table 4 and Table 5.

### 3.1. Malocclusion and Posture

Initially, we examined studies that investigated the relationship between malocclusion and overall posture, encompassing both general posture and specifically focusing on back posture and related disorders. We successfully identified 10 articles exploring the connection between posture and malocclusion, which are presented in Table 2.

**Table 2:** The correlation between malocclusion and posture.

Year	Title	Authors	Objective	Methods	Conclusions
2014	Assessment of the connection between the bite plane and body posture in children and teenagers [24]	Anna Gogola, Edward Saulicz, Małgorzata Matyja, Paweł Linek, Andrzej Myśliwiec, Agata Tuczyńska, Dagmara Molicka	Attempt to compare the occlusion condition in groups of children with different body posture.	336 children (aged 8–14) were divided into groups with different body posture according to Kasperczyk’s point method. The comparison of the occlusion was performed with a scale by Emmerich-Popłatek.	Children with faulty posture present more intense malocclusions than children with a correct body posture.
2019	Evaluation of a relationship between malocclusion and idiopathic scoliosis in children and adolescents [25]	M Laskowska, D Olczak-Kowalczyk, M Zadurska, J Czubak, M Czubak-Wrzosek, M Walerzak, M Tyrakowski	The aim was to analyze the relationships between the prevalence and type of malocclusions, and the presence of idiopathic scoliosis, its location, and severity.	The study group consisted of 80 patients with idiopathic scoliosis and the control group of 61 healthy individuals. Standard standing long-cassette radiographs were taken of all of the patients in the idiopathic scoliosis group. Both groups underwent standard clinical dental examinations.	In children and adolescents with idiopathic scoliosis, there is a significantly higher incidence of malocclusions than in the control group.
2013	Frequency of malocclusions in association with body posture problems in a school-age population from the State of Mexico [26]	Norma Angélica Aguilar Moreno, Olga Taboada Aranza	The objective was to determine the frequency of malocclusions associated with posture problems in a population of school-age children from the State of Mexico.	A study was carried out on 375 students (6–12 years old). Clinical evaluation of malocclusions was performed following the Angle and WHO criteria for the evaluation of postural attitude considering categories by observing their spinal columns and possible alterations in their frontal and sagittal planes.	When malocclusions are shown in children of this age, they are frequently related to posture problems. This stage of life is important due to the fact that this is the period when the majority of morphological and functional changes occur.
2011	Do malocclusion and Helkimo Index ≥ 5 correlate with body posture? [27]	L Perillo, B Femminella, D Farronato, T Baccetti, L Contardo, G Perinetti	The aim was to investigate whether malocclusal traits and having a Helkimo Index ≥ 5 show detectable correlations with body posture alterations in children and young adults.	A total of 1178 (11–19 years old) subjects were examined. Dental occlusion assessment included the following: overbite, overjet, posterior crossbite, scissor bite, mandibular crowding, and dental class. Body posture assessments were performed through static analyses of body inclination and trunk asymmetry, and according to the dynamic Fukuda stepping test.	Potential body postural effects associated with malocclusion or Helkimo Index ≥ 5 appear to be of little relevance. In subjects positive for either malocclusion or Helkimo Index ≥ 5, dental treatments should not include prevention or treatment of postural imbalance.
2010	Dental malocclusion and body posture in young subjects: a multiple regression study [28]	Giuseppe Perinetti, Luca Contardo, Armando Silvestrini-Biavati, Lucia Perdoni, Attilio Castaldo	The aim was to investigate whether malocclusal traits correlate with body posture alterations in young subjects to determine possible clinical applications.	122 subjects, (10–16 years old) were enrolled. A dental occlusion assessment included a phase of dentition, molar class, overjet, overbite, anterior and posterior crossbite, scissor bite, mandibular crowding, and dental midline deviation. Body posture was recorded using a vertical force platform.	The findings of the study do not support the existence of clinically relevant correlations between malocclusal traits and body posture.
2023	Association between posterior unilateral functional crossbite and asymmetrical spinal flexion: A prospective study [29]	Maria Grazia Piancino, Giada Matacena, Umberto Garagiola, Farhad B Naini, Alessandro Tortarolo, David Wertheim	The aim was to investigate whether the presence of UPC with functional shift is associated with reverse chewing pattern and altered spine flexion.	38 children with unilateral posterior crossbite (7–9 years old) had measurements before and after treatment. Patients with UPC and a control group were recorded when chewing using a Kinesiograph, and spine alignment was assessed.	This study suggests the association between unilateral posterior crossbite and asymmetrical flexion of the spine, increased on the crossbite side, as well as with asymmetrical chewing patterns.
2023	Malocclusion and Scoliosis: Is There a Correlation? [30]	Sabina Saccomanno, Stefano Saran, Licia Coceani Paskay, Nicola Giannotta, Rodolfo Francesco Mastrapasqua, Alessio Pirino and Fabio Scoppa	The objective was to assess the relationship between scoliosis and malocclusion.	A total of 646 patients were enrolled (447 with scoliosis and 199 without) to answer an anonymous questionnaire. They had dental and orthopedic examinations. Participants were given a bilingual survey composed of 13 questions formulated specifically for this study, using Google Forms (Google LLC, Mountain View, CA, USA).	There might be an important connection between malocclusion and scoliosis; dentists and orthopedists have to check for the probable presence of both pathologies to avoid a severe progression.
2019	Correlations between Malocclusion and Postural Anomalies in Children with Mixed Dentition [31]	Sergio Sambataro, Salvatore Bocchieri, Gabriele Cervino, Rosario La Bruna, Alessandra Cicciù, Marcella Innorta, Benedetto Torrisi, Marco Cicciù	The aim was to investigate the possible relationship between malocclusion and body posture anomalies.	127 children with mixed dentition were involved. Examination of oral cavity by an orthodontist—molar and canine relationship, crossbite, lower middle-line deviation, and centric relation considering mono or bilateral contacts in centric relation. Orthopedic examination by an orthopedist—scoliosis, false scoliosis or paramorphism, kyphosis, and lordosis.	There is a correlation between scoliosis and malocclusions on the transversal plane but not on the sagittal plane, and the presence of these types of malocclusions imposes a postural evaluation of the patient by the orthodontist.
2015	Relationships between Malocclusion, Body Posture, and Nasopharyngeal Pathology in Pre-Orthodontic Children [32]	Šidlauskienė M, Smailienė D, Lopatienė K, Čekanauskas E, Pribuišienė R, Šidlauskas M.	The aim was to examine the relationship between the type of malocclusion, body posture, and nasopharyngeal obstruction in children aged 7–14 years.	94 patients (7–14 years old) passed an examination by the orthodontist (study model and cephalometric radiograph analysis), orthopedic surgeon (body posture examined from the front, side, and back), and otorhinolaryngologist (anterior and posterior rhinoscopy and pharyngoscopy) in a blind manner.	There was a significant association between the sagittal position of the mandible (SNB angle) and a kyphotic posture; kyphotic posture was significantly more common among patients with nasopharyngeal obstruction.
2013	Clinical association between teeth malocclusions, wrong posture and ocular convergence disorders: an epidemiological investigation on primary school children [33]	Armando Silvestrini-Biavati, Marco Migliorati, Eleonora Demarziani, Simona Tecco, Piero Silvestrini-Biavati, Antonella Polimeni and Matteo Saccucci	The aim was to investigate the incidence of dental malocclusions together with posture and eye convergence disorders.	605 children underwent dental/occlusal (crossbite, midline deviation with a mandibular shift, bad habits, and deep or open bite), orthoptic and postural examination (frontal and lateral inspection, investigation during trunk flexion and ambulation, any asymmetry in the lower limbs).	About 13% of children showed a pathological gait and, among them, vertical anomalies of occlusion (deep bite or open bite) were prevalent compared to the other occlusal defects. The vertical dimension of occlusion revealed a slight relationship with the proper dominant eye.

### 3.2. Malocclusion and Cervical Vertebral Column Morphology and Head Posture

A total of six scholarly articles were identified, focusing on the intricate interrelation among the cervical vertebral column, head posture, and malocclusion. These articles were dedicated to unraveling the nuanced associations and potential correlative patterns that exist within the intricate biomechanical dynamics of the craniofacial complex, cervical morphology, and the alignment of the head in the context of malocclusive conditions, as summarized in Table 3.

**Table 3:** The correlation between malocclusion and cervical vertebral column morphology and head posture.

Year	Title	Authors	Objective	Methods	Conclusions
2011	Cervical vertebral column morphology related to craniofacial morphology and head posture in preorthodontic children with Class II malocclusion and horizontal maxillary overjet [34]	Torill Arntsen and Liselotte Sonnesen	The aims were to compare the morphology of the cervical column in a group of children with skeletal horizontal maxillary overjet with a group of children with dentoalveolar horizontal maxillary overjet and analyze associations between the morphology of the cervical column, craniofacial dimensions, and head posture in both groups.	213 profile radiographs were systematically selected and divided into 2 groups: skeletal (99 patients aged 7–15) and dentoalveolar overjet (114 patients aged 7–15).	Deviations in the cervical vertebral column morphology occurred significantly more often in the skeletal overjet group (28%) compared with the dentoalveolar overjet group (17%). Fusion anomalies were associated with a large sagittal jaw relationship, retrognathia of the jaws, large inclination of the jaws, and extended head posture. Furthermore, a partial cleft was significantly associated with a large cranial base angle.
2010	Posture and Posterior Crossbite in Oral and Nasal Breathing Children [35]	Jecilene Rosana Costa, Silvia Regina Amorim Pereira, Shirley S N Pignatari, Luc Louis Maurice Weckx	The aim was to evaluate the prevalence of posterior crossbite in a group of oral- and nasal-breathing children and associate the type of bite with the head and cervical spine posture.	98 children (9–12 years old) were submitted to a clinical otorhinolaryngologic evaluation.	Most of the children, either oral or nasal breathers, did not present crossbites. The type of head posture and cervical spine can vary independently of a posterior crossbite.
2019	Is Head Posture and Malocclusion Related? [36]	Aayush Kumar Garg, Tripti Tikku, Rohit Khanna, Rana Pratap Maurya, Kamna Srivastava, Sneh Lata Verma	The aim was to find the association between head posture and type of malocclusion using Digimizer software.	90 subjects (15–25 years old) were examined using Angle’s classification to evaluate the relationship between head posture and malocclusion.	The development of malocclusion has a multifactorial etiology, of which head posture is one of the factors resulting in malocclusion. This could be the reason that variabilities in postural angles determining head posture were seen in different malocclusion groups, but the difference was not significant and the correlation coefficient had less predictable values.
2021	Quantitative Analysis of Body Posture and Its Correlation with Cervical Posture in Various Malocclusions [37]	Shruthi Pradeep Priyanka Venkatasubramanian Ratna Parameswaran Devaki Vijayalakshmi	The aim was to investigate whether there are significant differences in posture in subjects with skeletal class I, class II, and class III malocclusion.	90 subjects (16–22 years old) with Angle class I, II, and III skeletal malocclusion. A customized force platform with pressure sensors was used for posture analysis.	Subjects with a class I skeletal base were found to have no or minimal alteration in body posture and cervical posture. Subjects with skeletal class II malocclusion werefound to have increased cervical curvature and a tendency to lean in the anterior direction with a forward extension of the head. Subjects with skeletal class III malocclusion were noted to have a decrease in cervical curvature in comparison to class I and class II skeletal base and to have a tendency to lean in the posterior direction with backward flexion of the head.
2019	Cervical Posture and Skeletal Malocclusions—Is there a Link? [38]	Sanam Tauheed, Attiya Shaikh, Mubassar Fida	The aim was to determine cervical posture in different skeletal sagittal malocclusions and to assess whether a correlation existed between cervical posture and skeletal relationships.	63 subjects (aged 11–22) were categorized into skeletal class I, II, and III. Cervical inclination was assessed.	Skeletal sagittal jaw relationships differ for cervical postures, especially cervical curvature. Skeletal class III subjects have straighter cervical columns than skeletal Class I. Curvature of the cervical column correlates with sagittal jaw relations.
2016	Relationship between head posture and parameters of sagittal position and length of jaws [39]	Vladanka Vukicevic, Dorde Petrovic	The aim was to examine the relationship between the head posture and parameters of the sagittal position and length of the jaws.	90 subjects (aged 8–14) had lateral cephalograms made and the parameters were analyzed using the Onyx Ceph program.	Class II patients have the highest value of the craniocervical angle, i.e., the greatest head extension in relation to the cervical spine. The positive correlation between the value of the craniocervical angle and the upper jaw length and a negative correlation between the value of the craniocervical angle and the lower jaw length can contribute to the occurrence of class II malocclusion.

### 3.3. Malocclusion and Podal System

This section explored recent scientific studies investigating the relationship between dental malocclusion, foot posture, and body balance in pediatric populations, shedding light on potential correlations and implications for postural health. The research works are summarized in Table 4.

**Table 4:** The correlation between malocclusion and podal system.

Year	Title	Authors	Objective	Methods	Conclusions
2021	Dental Malocclusion and Its Relation to the Podal System [40]	María E. Cabrera-Domínguez, —Reyes, Manuel Pabón-Carrasco, Ana J. Pérez-Belloso, Manuel Coheña-Jiménez, and Antonio F. Galán-González	The aim was to verify whether there is a relationship between dental occlusion and the podal system.	409 children (8–14 years old) were analyzed according to Angle’s classification.	A significant correlation was observed for the FPI points on the left foot and the scaphoid height on the right foot (*p* < 0.001). A predominance of anteriority of the center of gravity was found in subjects with Angle’s class II malocclusion. In those with Angle’s class I and III malocclusion, the center of gravity was in a more posterior location.
2018	Relationship between foot posture and dental malocclusions in children aged 6 to 9 years A cross-sectional study [41]	Ana Marchena-Rodríguez, Noelia Moreno-Morales, Edith Ramírez-Parga, María Teresa Labajo-Manzanares, Alejandro Luque-Suárez, Gabriel Gijon-Nogueron	The aim was to determine the association between foot posture and dental malocclusions in the anteroposterior plane in children.	Qualified personnel conducted a podiatric and dental examination of 189 children (6–9 years old).	The Clarke angle tends to decrease as Angle classification increases from class I to III, whereas the FPI is greater as Angle classification increases from class I to III. None of the study participants had a supinated foot in association with Angle class III, while approximately 50% of the pronated feet were associated with Angle class III.
2020	Influence of Dental Malocclusion on Body Posture and Foot Posture in Children: A Cross-Sectional Study [42]	Ana Juana Pérez-Belloso, Manuel Coheña-Jiménez, Maria Eugenia Cabrera-Domínguez, Antonio Francisco Galan-González, Antonia Domínguez-Reyes, Manuel Pabón-Carrasco	The objective was to evaluate if the features of dental malocclusion are correlated with body posture alterations at the lower limb level.	The study involved 289 children (8–14 years).	A direct relationship is not found between the stomatognathic system and the structures of the lower limb (hip, knee, and foot). Having compared the dental classification with the baricentre, significant data were found related to the contact surface, especially in the plantigrade phase, and fundamentally with the center of gravity. A predominance of the anteriority of the center of gravity in Angle’s class II is revealed.
2023	Dental Malocclusion in Mixed Dentition Children and Its Relation to Podal System and Gait Parameters [43]	Dorota Różańska-Perlińska, Jarosław Jaszczur-Nowicki, Dariusz Kruczkowski and Joanna Magdalena Bukowska	The purpose of this study was to confirm or deny the correlations between body posture and malocclusion.	76 patients (12–15 years old) were divided into two groups: without malocclusion and with malocclusion.	There is a correlation between the presence of stomatognathic disorder and gait cycle parameters.
2023	Changes in Gait Parameters and the Podal System Depending on the Presence of a Specific Malocclusion Type in School-Age Children [44]	Dorota Różańska-Perlińska, Jarosław Jaszczur-Nowicki, Łukasz Rydzik, Jacek Perliński and Joanna M. Bukowska	The aim was to analyze the relationship between various malocclusion types and gait parameters, the distribution of foot pressure on the ground, and body balance.	155 patients (aged 12–16) were divided into groups according to Angle’s classification.	There was a relationship between malocclusion and step duration. In canine class II, a relationship was noticed between the length of the left and right steps. There were no significant relationships between the pressure on the forefoot, midfoot, or heel area, and malocclusion. There was a significant relationship between the projection of the body’s center of gravity on the left foot and dental disorders in patients with a deep bite.

### 3.4. Malocclusion and Gait Parameters

In this part of the study, we dealt with four important research studies, each shedding light on various aspects of this complex association. A compilation of studies focused on the relationship between malocclusion and gait parameters is presented in Table 5.

**Table 5:** The correlation between malocclusion and gait parameters.

Year	Title	Author	Objective	Method	Conclusions
2023	Dental Malocclusion in Mixed Dentition Children and Its Relation to Podal System and Gait Parameters [43]	Dorota Różańska-Perlińska, Jarosław Jaszczur-Nowicki, Dariusz Kruczkowski and Joanna Magdalena Bukowska	The purpose of this study was to confirm the presence or absence of correlations between body posture and malocclusion.	76 patients (12–15 years old) were divided into two groups without malocclusion and with malocclusion, using Angle’s classification.	There is a correlation between the presence of stomatognathic disorder and gait cycle parameters.
2023	Changes in Gait Parameters and the Podal System Depending on the Presence of a Specific Malocclusion Type in School-Age Children [44]	Dorota Różańska-Perlińska, Jarosław Jaszczur-Nowicki, Łukasz Rydzik, Jacek Perliński and Joanna M. Bukowska	The aim was to analyze the relationship between various malocclusion types and gait parameters, the distribution of foot pressure on the ground, and body balance.	155 patients (aged 12–16) were divided into groups according to Angle’s classification.	There was a relationship between malocclusion and step duration. In canine class II, a relationship was noticed between the duration of the left and right steps. There were no significant relationships between the pressure on the forefoot, midfoot, or heel area, and malocclusion. There was a significant relationship between the projection of the body’s center of gravity on the left foot and dental disorders in patients with a deep bite.
2013	Clinical association between teeth malocclusions, wrong posture and ocular convergence disorders: an epidemiological investigation on primary school children [33]	Armando Silvestrini-Biavati, Marco Migliorati, Eleonora Demarziani, Simona Tecco, Piero Silvestrini-Biavati, Antonella Polimeni and Matteo Saccucci	The aim was to investigate the incidence of dental malocclusions together with posture and eye convergence disorders.	605 children underwent dental/occlusal, orthoptic, and postural examinations.	About 13% of children showed a pathological gait and, among them, vertical anomalies of occlusion (deep bite or open bite) were prevalent with respect to the other occlusal defects. The vertical dimension of occlusion revealed a slight relationship with the proper dominant eye.

### 3.5. Risk of Bias

An adapted version of the Newcastle-Ottawa Scale was used to assess the risk of bias in cross-sectional studies. Of the 24 studies examined, 15 (62.5%) were found to have a moderate risk of bias, while the remaining 9 (37.5%) had a low risk. A majority (62.5%) of the studies included in the analysis exhibited selection bias, while 50% of them had inadequate sample size and insufficient power, 59% exhibited performance bias, 25% exhibited detection bias, and 10% exhibited information bias (Table 6).

The overall quality and strength of the evidence based on the revised criteria of the Cochrane Back and Neck Pain Group are reported in Table 7.

## 4. Discussion

It was found that the optimal approach for analyzing the included articles would involve categorizing them into four groups depending on their contents:

1. Malocclusion and posture (in a general sense, back posture, back disorders).
2. Malocclusion and cervical vertebral column morphology and head posture.
3. Malocclusion and the podal system and foot posture.
4. Malocclusion and gait parameters.

### 4.1. Malocclusion and Posture

Understanding the intricate relationship between posture and malocclusion is crucial for comprehensive healthcare, particularly in pediatric populations. In this part, we analyzed 10 articles that dealt with various aspects of this correlation, exploring connections, treatment outcomes, and associated factors.

The study by Gogola et al., involving 336 children aged 8 to 14, revealed a noteworthy correlation between faulty posture and more severe malocclusions. It employed Emmerich-Popłatek’s scale for occlusion quality assessment and Kasperczyk’s method for posture classification, providing insights into the interplay between dental occlusion and body posture [24]. Focusing on Angle class II patients, the retrospective examination by Klostermann et al. suggested that overjet reduction during early orthodontic treatment may be associated with detectable effects on pelvic torsion, yet the authors concluded there were no significant differences in the back and posture patterns after early orthodontic treatment with removable appliances. Changes in body posture parameters have been analyzed using raster stereographic photogrammetry, emphasizing the importance of considering orthodontic interventions in relation to posture dynamics [45]. In the prospective longitudinal study by Laskowska et al. involving 80 scoliosis patients and 61 controls, a higher incidence of malocclusions was observed in children with idiopathic scoliosis. The most common malocclusion in children and adolescents with idiopathic scoliosis were: distoclusion and cross bites, asymmetric Angle class and canine class, dental anomalies, and no coincidence of the maxillary dental midline and the mandibular dental midline. The research underscored the need for a comprehensive approach to address both spinal and dental issues [25]. A randomized clinical trial by Lippold et al. with 80 patients demonstrated that early orthodontic treatment for unilateral posterior crossbite did not negatively impact postural parameters. No clinically relevant differences were found between therapy and control groups, emphasizing the safety of the orthodontic protocol employed [46]. The study by Mason et al. involving 41 patients evaluated the effects of rapid palatal expansion (RPE) on posture and gait. While RPE significantly improved posture during gait, static posture remained unaffected. The study underscored a detectable correlation between dental occlusion and body posture, emphasizing the benefits of RPE [47]. Conducted on 375 school-age children, the study by Moreno et al. identified a frequent relationship between malocclusions and posture problems. The research highlighted the importance of monitoring posture and occlusion during this crucial developmental period [26]. Involving 1178 participants, the investigation by Perillo et al. suggested that malocclusion or a Helkimo Index ≥ 5 may have limited correlations with body-posture alterations in young people. The study advised against including postural imbalance prevention or treatment in dental interventions for these individuals [27]. Analyzing 122 participants, the study by Perinetti et al. challenged the existence of clinically relevant correlations between malocclusal traits and body posture. The findings questioned the utility of posturography as a diagnostic aid for dental malocclusion [28]. The study by Piancino et al. suggested an association between unilateral posterior crossbite and asymmetrical spine flexion, emphasizing the relevance of assessing chewing patterns in understanding the link between malocclusion and spinal deviations [29]. With 646 participants, the study by Saccomanno et al. supported the connection between malocclusion and scoliosis. It underscored the importance of early assessment by dentists and orthopedists to prevent severe progression, potentially requiring significant therapy or surgery [30]. Investigating 127 children, the study by Sambataro et al. found a correlation between scoliosis and malocclusions on the transversal plane but not on the sagittal plane. It highlighted the need for orthodontic evaluation in patients with malocclusions [31]. Examining 94 patients aged 7–14, the study by Šidlauskienė et al. established a significant association between the sagittal position of the mandible and kyphotic posture. Nasopharyngeal obstruction was identified as a factor influencing body posture in these children [32]. Conducted on 605 primary school children, the epidemiological investigation by Silvestrini-Biavato et al. revealed that about 13% of children with pathological gait exhibited vertical anomalies of occlusion, emphasizing the need for a multidisciplinary medical approach to treatment [33]. The study by Smailienė et al. involving 23 children suggested that orthodontic treatment with twin-block appliances contributed to the straightening of the back profile and a statistically significant reduction in posture-related measurements. However, it acknowledged that these changes might be influenced by physiological growth [48].

The investigation into the correlation between malocclusion and posture has spurred substantial research, contributing valuable insights into the potential interdependence of these physiological aspects. The majority of the articles (10 out of the 14 articles) affirmed a discernible association between malocclusion and posture. The studies employed diverse methodologies, including gait analysis, posturographic examination, and clinical assessments. One research study (“Gait and Posture Analysis in Patients with Maxillary Transverse Discrepancy, Before and After RPE”, by Martina Mason et al.) confirmed the relation between treatment of malocclusion and posture in gait but found no evidence of changes in static posture after therapy [47].

However, four articles presented a more nuanced stance, suggesting that potential correlations, particularly those related to malocclusal traits, may have limited clinical relevance.

In the field of spinal health, two articles stand out for their exploration of the relationship between malocclusion and scoliosis. “Evaluation of a Relationship Between Malocclusion and Idiopathic Scoliosis in Children and Adolescents” [25] and “Malocclusion and Scoliosis: Is There a Correlation?” [30] both contribute to the understanding that a potential connection exists between malocclusion and scoliosis, emphasizing the importance of interdisciplinary examination.

### 4.2. Malocclusion and Cervical Vertebral Column Morphology and Head Posture

The findings of Arntsen and Sennesen revealed a pronounced association between deviations in cervical vertebral morphology and sagittal jaw relationships, highlighting a pivotal connection between craniofacial dimensions and head posture [34]. The study by Bardellini et al. underscored the reciprocal influence, affirming that correct dental occlusion positively impacts body posture, particularly the head’s physiological extension and balanced weight distribution [49]. In the research by Costa et al., it is worth emphasizing the independence of head and cervical spine posture in influencing posterior crossbite, irrespective of breathing patterns [35]. On the other hand, Garg et al. recognized the multifactorial etiology of malocclusion, demonstrating variability in postural angles across different types of malocclusions without statistical significance. Yet, the research focused on a demographic that was more advanced in age (adolescents and young adults) compared to other studies [36]. Ting et al. illuminated the compensatory mechanism of poor cervical spine posture in skeletal class malocclusion, necessitating a nuanced approach to sagittal dysfunction correction [50]. Pradeep et al. underlined the intricate correlation between cervical and body posture, with class II malocclusion notably linked to increased cervical curvature and anterior head inclination. The study involved an elderly population, which is older than the participants in the other research projects [37]. The investigations by Tauheed revealed significant differences in cervical curvature among skeletal sagittal malocclusions, particularly highlighting straighter cervical columns in class III subjects [38]. Vukicevic and Petrovic proved that class II malocclusion was a significant factor in increased craniocervical angle, contributing to maxillary prognathism and reduced mandibular prognathism [39].

Upon meticulous review of the six included articles, the majority (five out of six) lean towards confirming the relationship between malocclusion and cervical column morphology and head posture. These five studies collectively emphasize the intricate nature of this relationship, acknowledging the nuanced associations between craniofacial structures and body positioning. While some variations exist in the methodologies and focal points of these investigations, a consensus emerged regarding the multifactorial nature of these interactions, emphasizing the need for further interdisciplinary research. One remaining study did not support the thesis that there was a correspondence between head/cervical column posture and malocclusion. The research by Garg et al. found that the development of malocclusion is multifactorial, with head posture being a contributing factor. Despite observing variability in postural angles across malocclusion groups, the study suggests that these differences lacked statistical significance, and correlation coefficients yielded less predictable values, indicating a complex relationship between head posture and types of malocclusions [36]. Results by Costa et al. indicated that a majority of children, irrespective of breathing patterns, did not exhibit crossbite. The study highlighted that the presence of posterior crossbite can vary independently of head posture and cervical spine positioning, emphasizing the intricate relationship between these factors in dental and orthodontic considerations [35].

### 4.3. Malocclusion and the Podal System

Elena Bardellini et al. highlighted the positive effects of correct dental occlusion on podalic support and homogeneous weight distribution on both feet [49]. María E. Cabrera-Domínguez et al. explored the correlation between dental occlusion and the podal system in 409 children. Significant correlations were observed for FPI points and scaphoid height concerning dental malocclusions. The study identified anteriority of the center of gravity in Angle’s class II malocclusion and posterior situations in Angle’s class I and III, suggesting a moderate relationship that warrants further comprehensive studies [40]. Ana Marchena-Rodríguez et al. conducted a cross-sectional study on 189 children, revealing a potential relation between Clarke angle, FPI, and dental malocclusion. The Clarke angle tended to decrease, and FPI increased with Angle classification from class I to III. This study provided insights into the interplay between foot posture and dental malocclusions in children [41]. Ana Juana Pérez-Belloso et al. performed a multicenter cross-sectional study involving 289 children. The research demonstrated a strong correlation between lower limb biomechanics and a moderate relationship between dental malocclusion and the center of gravity. The study emphasized the intricate connection between stomatognathic and lower limb structures, particularly in plantigrade phases [42]. Dorota Różańska-Perlińska et al. explored correlations between body posture, malocclusion, and gait parameters in 76 patients. The study used a pedobarographic mat, Wiva^®®^ Science, and Kineod 3D for comprehensive analysis, and confirmed a correlation between stomatognathic disorders and gait cycle parameters [43]. Another article by these authors investigated relationships between types of malocclusions and gait parameters in 155 school-age children. The study found significant associations between dental abnormalities and step length, as well as the projection of the body’s center of gravity on the left foot in patients with a deep bite [44]. The reviewed articles collectively underscored the intricate connections between dental malocclusion, foot posture, and body balance in children. While acknowledging varying degrees of correlation, these studies highlighted the need for further extensive research to comprehend the nuanced interplay between stomatognathic and lower limb structures, providing valuable insights for holistic treatment approaches.

### 4.4. Malocclusion and Gait Parameters

The research by Mason et al., conducted to analyze 41 participants aged 6 to 12, focuses on the effects of rapid palatal expansion (RPE) on posture, especially during gait. The study used gait analysis and posturographic examination to categorize participants into controls, participants with a unilateral posterior crossbite, and those with maxillary transverse discrepancy. The key finding was a significant improvement in posture during gait, specifically in the cranio-caudal direction, following the correction of transverse arch dimensions. Intriguingly, while dynamic posture sees improvement, static posture remains unaffected. Importantly, this study established a discernible correlation between dental occlusion and body posture, underlining an additional benefit associated with RPE [47]. Różańska-Perlińska et al., in “Dental Malocclusion in Mixed Dentition Children and Its Relation to Podal System and Gait Parameter”, focused on the correlation between dental malocclusion and gait parameters in mixed dentition children. Seventy-six participants, aged 12–15, were categorized into groups based on the presence or absence of malocclusion. The comprehensive methodology included the use of a pedobarographic mat for foot force distribution analysis, a Wiva^®^ Science diagnostic system for gait analysis, and Kineod 3D for posture analysis. The findings revealed a significant correlation between the presence of stomatognathic disorders and various parameters of the gait cycle, highlighting the intricate interplay between dental occlusion and gait dynamics [43]. Further research made by Różańska-Perlińska et al., “Changes in Gait Parameters and the Podal System Depending on the Presence of a Specific Malocclusion Type in School-Age Children”, encompassed 155 participants aged 12–16. The study examined the nuances of types of malocclusions and their impact on gait parameters. Stratifying participants into types of malocclusions such as Angle’s class II, canine class II, and overbite, the research employed advanced methodologies including the Wiva^®^ Science sensor and an E.P.S R/1 pedobarographic mat for the analysis of gait and foot forces. Noteworthy correlations were found between dental abnormalities within malocclusion and step duration, with specific attention to the pronounced associations in children with canine class II malocclusion [44]. Silvestrini-Biavati et al. conducted a comprehensive examination of 605 primary school children. This epidemiological investigation explored the association between teeth malocclusions, posture, and ocular convergence disorders. The study integrated dental/occlusal, orthoptic, and postural evaluations and found that 13% of children exhibited pathological gait. Vertical occlusal anomalies, particularly deep bite or open bite, were prevalent among those with pathological gait. The study advocated for a multidisciplinary medical approach, acknowledging the intricate interconnections between postural, orthoptic, osteopathic, and occlusal variables [33].

Collectively, these research studies have contributed substantially to the understanding of the multifaceted relationship between malocclusion, dental occlusion, and gait parameters in pediatric populations. While each study provides unique insights, similarities include the emphasis on gait analysis, posturography, and the intricate correlations discovered. The divergences in the impact of interventions or conditions, such as RPE or specific types of malocclusions, underscore the intricate nature of these interrelated health domains. Moreover, the call for a multidisciplinary approach, particularly highlighted in the epidemiological investigation, emphasizes the complexity of diagnosis and treatment in this domain. As our understanding deepens, these studies collectively pave the way for more nuanced and effective interventions in the field of pediatric malocclusion and gait dynamics.

### 4.5. Limitations

One of the limitations of this systematic review is the retrospective registration of the protocol in the PROSPERO database, which may have affected the transparency and potential bias in the analysis. Additionally, the quality of evidence was not assessed using the GRADE methodology, which limits the ability to accurately evaluate the strength and certainty of the results. Therefore, the findings of this study should be interpreted with caution.

## 5. Conclusions

The majority of studies on malocclusion and posture have confirmed a discernible association between malocclusion and posture. Various methodologies, including gait analysis, posturographic examination, and clinical assessments, have been employed to explore this relationship. Notably, a study on the effects of rapid palatal expansion (RPE) on posture during gait indicated a significant improvement in dynamic posture and emphasized the potential benefits of orthodontic interventions in enhancing overall body balance.

The majority of the studies focused on the relationship between malocclusion, cervical vertebral column morphology, and head posture; emphasized the intricate nature of this relationship; and acknowledged the nuanced associations between craniofacial structures and body positioning.

The studies exploring the relationship between malocclusion, foot posture, and body balance highlighted the positive effects of correct dental occlusion on podalic support and homogeneous weight distribution on both feet.

The studies dealt with various aspects of the complex association between malocclusion and gait parameters in pediatric populations and collectively have contributed substantially to the understanding of the relationship between malocclusion, dental occlusion, and gait dynamics.

The present systematic review provides a comprehensive overview of the current state of knowledge on the correlations between malocclusion and various physiological parameters in children. The findings underscore the need for further interdisciplinary research, considering the multifactorial nature of these relationships, and pave the way for more nuanced and effective interventions in the field of pediatric malocclusion and associated health dynamics.

## Figures and Tables

**Table 1:** Inclusion and exclusion criteria used for the analysis.

Inclusion Criteria	Exclusion Criteria
Consistent with the subject of the study	Investigations deviating from the designated thematic scope
Year of publication: 2010–2023	Articles concerning systematic reviews
Children and adolescent population examined	Articles concerning adult populations
English language	
No reviews taken into analysis	
Observational studies (cross-sectional, case-control, longitudinal)	

**Table 6:** Risk of Bias Assessment of Cross-Sectional Studies (Newcastle Ottawa Scale, adapted version).

First Author	Selection Bias	Performance Bias		Detection Bias		Information Bias		Total Score	Quality
	A	B	C	D	E	F	G		
Malocclusion and posture									
Anna Gogola [24]	2	3	1	1	3	2	2	14/21	low risk
Laskowska [25]	1	1	1	1	3	3	2	12/21	moderate
Angélica [26]	2	3	2	2	3	3	2	17/21	low risk
Perillo [27]	1	3	1	2	3	2	2	14/21	low risk
Perinetti [28]	1	1	2	2	2	2	2	12/21	moderate
Piancino [29]	1	0	1	2	3	3	2	12/21	moderate
Saccomanno [30]	2	3	1	2	3	3	2	16/21	low risk
Sambataro [31]	1	0	1	2	3	2	2	12/21	moderate
Šidlauskienė [32]	1	0	0	2	3	2	2	10/21	moderate
Silvestrini-Biavati [33]	1	3	1	2	3	3	2	15/21	low risk
Malocclusion and cervical vertebral column morphology and head posture									
Arntsen [34]	1	2	1	2	3	3	2	14/21	low risk
Costa [35]	1	0	0	2	3	3	2	11/21	moderate
Garg [36]	1	0	0	1	3	3	2	10/21	moderate
Pradeep [37]	1	0	1	2	2	3	2	11/21	moderate
Tauheed [38]	1	0	1	2	2	3	2	11/21	moderate
Vukicevic [39]	1	1	1	2	2	3	2	12/21	moderate
Malocclusion and the podal system									
Domínguez [40]	1	3	1	2	3	3	2	15/21	low risk
Rodríguez [41]	1	2	1	1	2	3	2	12/21	moderate
Belloso [42]	1	2	1	2	3	3	2	14/21	low risk
Perlińska [43]	1	1	1	2	2	3	2	12/21	moderate
Perlińska [44]	1	2	1	2	2	3	2	13/21	moderate
Malocclusion and gait parameters									
Perlińska [43]	1	1	1	2	2	3	2	12/21	moderate
Perlińska [44]	1	2	1	2	2	3	2	13/21	moderate
Silvestrini-Biavati [33]	1	3	1	2	3	3	2	15/21	low risk

Note: low risk of bias: score from 14/21 to 21/21; moderate risk of bias: score from 7/21 to 13/21; high risk of bias: score from 0/21 to 6/21; A = Is the source population (cases, controls, cohorts) appropriate and representative of the population of interest?; B = Is the sample size adequate and is there sufficient power to detect a meaningful difference in the outcome of interest?; C = Did the study identify and adjust for any variables or confounders that may influence the outcome?; D = Did the study use appropriate statistical analysis methods relative to the outcome of interest?; E = Are there any missing data and did the study handle this accordingly?; F = Is the methodology of the outcome measurement explicitly stated and is it appropriate?; G = Is there an objective assessment of the outcome of interest?

**Table 7:** Modified Cochrane Back and Neck Pain Group criteria for the overall level of evidence based on cross-sectional studies.

Outcome	Number of Studies	Number of Participants	Level of Evidence
Malocclusion (Association with)			
posture	10	3601	strong (positive association)
cervical vertebral and head posture	6	644	moderate evidence (positive association)
podal system	5	1118	strong (positive association)
gait parameters	3	836	moderate evidence (positive association)

Strong evidence: consistent findings in two or more studies with low risk of bias (score from 14 to 21 on NOS); moderate evidence: consistent findings in one study with low risk of bias or two or more studies with moderate risk of bias (score from 8 to 13 on NOS); limited evidence: consistent findings in one or more studies with high risk of bias (score from 0 to 7 on NOS) or one study with moderate risk of bias; no evidence: no published studies found; conflicting evidence: inconsistent or contradictory findings within a quality level.

## Data Availability

The data presented in this study are available on request from the corresponding author.
